# Structural Insights into HLA-DQ–Associated Susceptibility to Celiac Disease Through an Integrated Genetic and In Silico Approach in a Sardinian Population

**DOI:** 10.3390/genes17020145

**Published:** 2026-01-28

**Authors:** Faustina Barbara Cannea, Daniela Diana, Rossano Rossino, Alessandra Padiglia

**Affiliations:** 1 Department of Life and Environmental Sciences (DiSVA), Biomedical Section, University of Cagliari, Cittadella Universitaria di Monserrato, 09042 Monserrato, Italy; faustinab.cannea@unica.it; 2 Department of Biomedical Sciences (DiSB), University of Cagliari, Cittadella Universitaria di Monserrato, 09042 Monserrato, Italy; d.diana6@studenti.unica.it; 3 Department of Biomedical Sciences and Public Health (DSMSP), University of Cagliari, AOU Presidio Microcitemico, Via Jenner, 09121 Cagliari, Italy; rossino40@gmail.com

**Keywords:** celiac disease, HLA class II, HLA-DQ2.5, HLA-DQ8, HLA-DQ2.2, HLA-DR/DQ haplotypes, Sardinian population, secondary structure prediction, PSIPRED, immunogenetics

## Abstract

Background: Celiac disease (CD) is a multifactorial autoimmune disorder strongly associated with specific HLA class II molecules, particularly HLA-DQ–encoding haplotypes. Although the genetic contribution of these loci is well established, the structural features accompanying allele-specific disease susceptibility remain incompletely explored. Methods: In this study, molecular HLA typing was integrated with in silico secondary structure analysis to examine the relationship between genetic predisposition and structural organization of HLA class II molecules in a Sardinian population. A total of 100 patients with CD and 100 healthy controls were genotyped for HLA-DR and HLA-DQ alleles, and allelic and haplotypic distributions were compared between groups. Secondary structure predictions were performed using PSIPRED on selected HLA class II alleles, focusing on groove-forming domains of HLA-DRB1 and HLA-DQA1. Results: CD patients showed a marked enrichment of the DR3–DQ2.5 haplotype, together with a population-specific predominance of DQ2.5 and a reduced contribution of DQ8. Secondary structure analysis of the HLA-DRB1 β1 domain revealed a largely conserved organization, with only modest allele-dependent variations. In contrast, comparative analysis of HLA-DQA1 identified localized differences within the α1 domain, with the DQ2.5 molecule displaying a more coherent secondary structure organization compared with the lower-risk DQ2.2 variant. Conclusions: By integrating genetic and in silico structural analyses, this study highlights that HLA-associated susceptibility to celiac disease reflects not only allele and haplotype distribution but also subtle, allele-specific features in the structural organization of peptide-binding regions. These findings provide a refined framework for interpreting HLA-DQ–mediated genetic risk and support the relevance of structural coherence as a complementary dimension in the assessment of disease susceptibility.

## 1. Introduction

CD is a chronic autoimmune enteropathy of the small intestine that develops in genetically predisposed individuals following the ingestion of gluten, a complex mixture of storage proteins present in cereals such as wheat, barley, rye, oats, spelt, and emmer wheat [1,2]. Genetic susceptibility to CD is primarily determined by specific alleles of the human leukocyte antigen (HLA) system, particularly the HLA-DQ2 and HLA-DQ8 haplotypes [3,4,5]. The central role of genetic factors is supported by family studies reporting a disease prevalence of approximately 10% among first-degree relatives and up to 30% in monozygotic twins [6,7,8]. Notably, about 90–95% of patients with CD express HLA-DQ2 molecules, whereas the remaining 5–10% carry HLA-DQ8 [9].

Nevertheless, the presence of these alleles alone is not sufficient to trigger disease onset; rather, it represents a necessary but not exclusive condition for the development of an autoimmune response to gluten in the presence of additional environmental and immunological factors. Recent advances in immunogenetics and structural immunology have highlighted the value of integrating genetic association data with structural analyses of HLA molecules to refine the interpretation of allele-specific disease susceptibility [10,11]. In this context, increasing attention has been directed toward understanding how subtle, allele-dependent structural features of HLA class II molecules may influence peptide accommodation and immune activation, providing an additional interpretative layer beyond classical genetic association.

The genes encoding HLA molecules are clustered within the major histocompatibility complex (MHC), located on the short arm of chromosome 6 (6p21.3), and share a conserved exon–intron organization across both class I and class II loci. Exons encode the primary structure of the polypeptide chains, whereas introns contain regulatory non-coding sequences [12,13]. The modular architecture of the HLA gene family underlies its extraordinary polymorphism, which is essential for the recognition of a wide repertoire of antigenic peptides. From an evolutionary perspective, HLA genes are thought to have arisen through duplication and rearrangement events from an ancestral genomic region, generating multiple functionally specialized loci involved in immune surveillance. Within this genomic framework, specific HLA class II haplotypes play a central role in genetic susceptibility to celiac disease, with important implications for HLA molecular typing in clinical practice [14].

### 1.1. HLA Class II Gene Organization and Genetic Background of CD

HLA class II genes encode heterodimeric glycoproteins composed of α and β chains, both of which are polymorphic and contribute to antigen presentation. The extracellular α1 and β1 domains form the core of the peptide-binding groove, followed by membrane-proximal domains, a transmembrane region, and a short cytoplasmic tail [13,15,16]. The HLA class II region includes the HLA-DR, HLA-DQ, and HLA-DP loci, which play a central role in adaptive immune responses.

Among these loci, HLA-DQ and HLA-DR are particularly relevant to CD susceptibility due to their strong haplotypic association within the MHC class II region and their coordinated contribution to antigen presentation. Genetic predisposition to CD is closely linked to the presence of HLA-DQ2 and HLA-DQ8 molecules, encoded by specific allelic combinations of HLA-DQA1 and HLA-DQB1 that present deamidated gliadin peptides to CD4^+^ T lymphocytes [1,3,5,9,12]. However, the distribution and expression of HLA-DQ alleles are strongly influenced by the HLA-DR background, which is in tight linkage disequilibrium with HLA-DQ [1,5,16,17,18].

**Figure 1:**
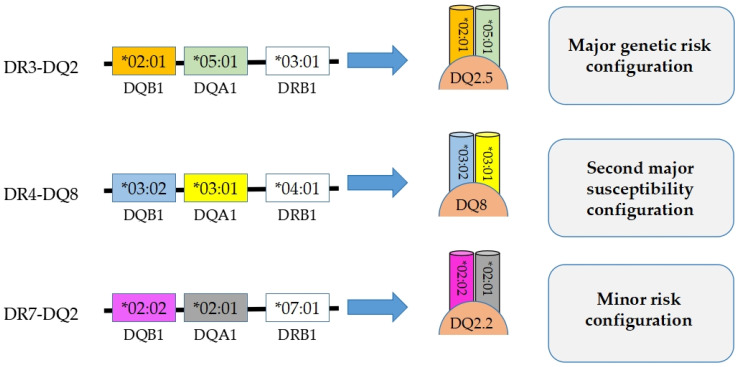
Conceptual schematic overview of major HLA-DR/DQ haplotypic configurations associated with CD susceptibility. The figure illustrates the most commonly reported HLA-DR/DQ haplotypes associated with different levels of genetic risk, including the major risk configuration DR3–DQ2.5, the second major susceptibility configuration DR4–DQ8, and the minor risk configuration DR7–DQ2.2. Risk categories are shown for illustrative purposes only and reflect established associations described in the literature, rather than quantitative estimates derived from the present dataset.

In this context, the HLA-DRB1*03:01–HLA-DQA1*05:01–HLA-DQB1*02:01 (DR3–DQ2.5) haplotype represents the major genetic risk configuration for CD, whereas HLA-DRB1*04:01–HLA-DQA1*03:01–HLA-DQB1*03:02 (DR4–DQ8) constitutes a second major susceptibility configuration, albeit less frequent [5,19]. In addition, the HLA-DRB1*07:01–HLA-DQA1*02:01–HLA-DQB1*02:02 (DR7–DQ2.2) haplotype is generally regarded as a minor-risk configuration [20,21]. This haplotypic organization defines a stable genetic framework within which allele-specific structural features of HLA-DQ molecules may contribute to variability in peptide-binding landscapes and immune recognition [10]. Although HLA-DR molecules do not directly bind gluten-derived peptides, they define a haplotypic context that conditions the presence and functional expression of disease-relevant HLA-DQ heterodimers. This strong linkage supports the inclusion of HLA-DR alleles in integrated analyses aimed at characterizing the genetic and structural background underlying CD susceptibility [1,5,22]. A schematic overview of the major HLA-DR/DQ haplotypic configurations associated with different levels of CD risk is provided in Figure 1 to support the genetic framework of the present study.

### 1.2. Structural Features of HLA-DR and HLA-DQ Molecules in CD

HLA class II molecules are non-covalent heterodimers whose α1 and β1 domains form an open-ended peptide-binding groove capable of accommodating peptides of variable length, typically ranging from 13 to 25 amino acids. This structural feature distinguishes HLA class II molecules from HLA class I molecules, which possess a closed peptide-binding groove and preferentially bind shorter peptides [23,24].

Structural studies have identified a series of binding pockets (P1–P9) within the class II peptide-binding groove that contribute to peptide accommodation and allelic specificity [25,26]. In HLA-DQ2 and HLA-DQ8 molecules, the physicochemical properties of these pockets favor the interaction with negatively charged residues present in deamidated gliadin peptides, thereby supporting CD4^+^ T-cell activation [27,28]. Although HLA-DR molecules do not directly bind gluten-derived peptides, their structural organization contributes to the haplotypic framework that conditions HLA-DQ expression and functional presentation within the MHC class II region.

Crystallographic analyses have provided detailed insights into peptide–HLA interactions at atomic resolution; however, such data are available for a limited number of allelic variants and do not fully capture population-level or allele-specific variability. Moreover, proper assembly and peptide loading of HLA class II molecules require the invariant chain (Ii, CD74), removal of the class II–associated invariant chain peptide (CLIP), and peptide exchange mediated by HLA-DM, underscoring the dynamic nature of antigen presentation beyond static binding interactions [29,30].

Consistent with their immunological relevance, histological and immunohistochemical studies have reported aberrant expression of HLA-DR and, to a lesser extent, HLA-DQ molecules in the intestinal epithelium of patients with CD, correlating with increased intraepithelial lymphocyte infiltration [31]. Together, these observations highlight the complexity of HLA-mediated antigen presentation and support the need for complementary approaches capable of addressing subtle, allele-dependent structural features that may contribute to HLA-associated susceptibility.

In this context, comparative analyses focusing on secondary structure organization remain relatively limited, despite their potential to provide a structural framework for interpreting genetic associations [32,33]. This gap supports the use of integrative strategies combining molecular HLA typing with in silico structural analyses to refine the interpretation of HLA-associated risk in celiac disease.

### 1.3. Immunopathogenesis of CD

Following dietary intake, gluten is partially digested into gliadin and glutenin fractions [34]. In genetically predisposed individuals, increased intestinal permeability, mediated in part by zonulin, allows gliadin peptides to cross the epithelial barrier and reach the lamina propria [35,36,37]. Here, tissue transglutaminase (tTG) catalyzes the deamidation of specific glutamine residues, converting them into glutamic acid and increasing peptide affinity for HLA-DQ2 and HLA-DQ8 molecules [38,39].

The resulting peptide–HLA complexes activate CD4^+^ T helper cells, predominantly of the Th1 phenotype, with contributions from additional T-cell subsets. This immune activation leads to cytokine release, epithelial apoptosis, villous atrophy, and crypt hyperplasia [3,40]. In parallel, B-cell activation results in the production of disease-specific autoantibodies, including anti-gliadin, anti-transglutaminase, anti-endomysium, and anti-deamidated gliadin peptide antibodies [41,42].

### 1.4. Aim of the Study

Although the presence of HLA-DQ2 and HLA-DQ8 molecules represents a necessary prerequisite for CD development, it is not sufficient to fully explain disease onset, highlighting the contribution of additional genetic, epigenetic, and environmental factors.

In this context, the present study aims to move beyond classical genetic association by integrating molecular HLA typing with in silico secondary structure analysis of HLA class II molecules. Specifically, we investigate whether allele- and haplotype-specific susceptibility to celiac disease is accompanied by subtle differences in the predicted secondary structure organization of groove-forming domains, with the goal of providing a structurally informed interpretation of HLA-associated risk.

Molecular HLA typing provides essential information on allele and haplotype distribution and represents the cornerstone for assessing genetic predisposition to celiac disease. However, when considered alone, genetic association studies do not fully explain why specific alleles confer different levels of disease risk. Conversely, structural analyses offer insights into the organization of peptide-binding regions but lack population-level context when not supported by genetic data. By integrating molecular and in silico structural approaches, the present study leverages the strengths of both methods while mitigating their individual limitations, enabling a more comprehensive and contextual interpretation of HLA-associated susceptibility to celiac disease in line with current immunogenetic research [32,33].

The remainder of the manuscript is organized as follows. Section 2 describes the study design, the characteristics of the study population, and the molecular and in silico methods employed. Section 3 presents the results of the genetic analyses and comparative secondary structure predictions, together with their integrated interpretation. Finally, Section 4 summarizes the main findings of the study and discusses their clinical applicability and broader implications for understanding HLA-associated susceptibility to celiac disease.

## 2. Materials and Methods

### 2.1. Ethical Approval and Informed Consent

The study was conducted in accordance with the principles of the Declaration of Helsinki and approved by the Comitato Etico Sardegna, established by Regional Decree No. 18 of 4 May 2023. Ethical approval was granted during the Committee meeting held on 27 May 2024 (Ethics Committee Report No. 41, protocol code ROMA23).

Written informed consent was obtained from all participants or, in the case of minors, from their parents or legal guardians, together with assent from minors when appropriate. All samples and data were processed in anonymized form, in compliance with current national and European regulations on data protection and good clinical practice.

### 2.2. Study Design

The study was structured into two complementary phases: (i) a molecular analysis aimed at genotyping HLA-DR and HLA-DQ susceptibility alleles, and (ii) an in silico secondary structure analysis of selected HLA class II molecules.

### 2.3. Study Population

The study included 100 patients with CD and 100 healthy controls from the Sardinian population, comprising children and adolescents aged 6–18 years. CD diagnosis was established based on positive serological testing, in accordance with current pediatric diagnostic guidelines. Control subjects had no clinical or serological evidence of CD. The study population was recruited within a relatively homogeneous genetic and geographic context, as all subjects were of Sardinian origin. Stratification by sex and the presence of comorbidities was not performed, as these variables were not expected to influence HLA class II allele distribution or the in silico secondary structure analyses, which were conducted on genomic DNA sequences. Accordingly, age and sex were not considered confounding variables for the genetic and structural investigations carried out in the present study.

Clinical severity scores and histological grading were not included, as the study was not designed to address genotype–phenotype correlations related to disease severity.

### 2.4. DNA Extraction and Quality Control

Genomic DNA was extracted from saliva samples using a commercial silica-based kit (Tissue DNA Purification Kit, EURx, Gdańsk, Poland), according to the manufacturer’s instructions. DNA concentration and purity were assessed using a NanoDrop spectrophotometer (Thermo Fisher Scientific, Waltham, MA, USA), and only samples with A260/A280 ratios between 1.8 and 2.0 were included in subsequent analyses.

### 2.5. HLA Genotyping by PCR–SSP

Molecular typing of HLA-DR and HLA-DQ loci was performed using PCR with sequence-specific primers (PCR–SSP) [43] and a commercial typing kit (Olerup SSP, Stockholm, Sweden). Reactions were set up according to the manufacturer’s protocol using 30–50 ng of genomic DNA per reaction. For each individual, 32 allele-specific PCR reactions were performed. PCR products were analyzed by agarose gel electrophoresis, and allele assignment was based on the presence or absence of specific amplification products using the reference tables provided by the manufacturer. Genotype interpretation was further supported by the Helmberg-SCORE™ software (Olerup SSP, Stockholm, Sweden), which enables automated reading and validation of amplification patterns against the reference database provided by the manufacturer. Each assay included a negative control to exclude contamination.

### 2.6. In Silico Analysis and Sequence Retrieval

For the in silico analyses, amino acid sequences of HLA class II alleles relevant to CD susceptibility, together with non-predisposing reference alleles, were retrieved from the IPD–IMGT/HLA database (https://www.ebi.ac.uk/ipd/imgt/hla/, accessed on 1 December 2025 ) [44]. The following HLA-DRB1 alleles were selected: HLA-DRB1*03:01 and HLA-DRB1*04:01 (predisposing alleles), and HLA-DRB1*10:01 and HLA-DRB1*11:01 (non-predisposing reference alleles). Linkage disequilibrium among predisposing HLA class II loci was considered, with particular focus on the associations between DQ2.5 (HLA-DQA1*05:01/HLA-DQB1*02:01) and HLA-DRB1*03:01, and between DQ8 (HLA-DQA1*03:01/HLA-DQB1*03:02) and HLA-DRB1*04:01 [45]. Based on genotyping results, individuals carrying predisposing alleles were selected as reference for subsequent structural analyses, in order to characterize allele-associated structural features. The full-length amino acid sequences of HLA-DR and HLA-DQ molecules retrieved from the IPD–IMGT/HLA database and used for the in silico analyses are provided in Supplementary Figure S1. Allele names are reported at the two-field resolution (e.g., HLA-DRB1*03:01), which uniquely defines the encoded protein sequence, according to IPD–IMGT/HLA nomenclature.

### 2.7. Secondary Structure Prediction

Secondary structure prediction was performed using PSIPRED v4.0 (http://bioinf.cs.ucl.ac.uk/psipred/, accessed on 1 December 2025) as the primary computational tool [46]. Protein FASTA sequences corresponding to selected HLA class II alleles were retrieved from the IPD–IMGT/HLA database and used as input for the analyses [44]. The set of alleles included key predisposing variants associated with CD (HLA-DRB1*03:01, HLA-DRB1*04:01, HLA-DQA1*05:01 [DQ2.5], and HLA-DQA1*02:01 [DQ2.2]), together with non-predisposing reference alleles (HLA-DRB1*10:01 and HLA-DRB1*11:01) for comparative purposes. PSIPRED integrates machine-learning algorithms with evolutionary profile information to predict α-helices, β-strands, and coil regions along protein sequences and was used as the primary tool for structural interpretation.

Comparative analyses were performed to identify conserved and variable secondary structure elements among the analyzed alleles. Structural interpretation focused on the peptide-binding groove of HLA class II molecules, with particular attention to the P1, P4, P6, and P9 pockets. Analyses were restricted to the α1 and β1 domains, corresponding approximately to residues 1–80 of the α chain and 1–90 of the β chain, which encompass the structural elements forming the peptide-binding groove. The relevance of these domains for pocket formation has been established by crystallographic studies [16,47]. Given the predominant contribution of the α chain to peptide-binding specificity and stability in DQ2.5 and DQ2.2 molecules, secondary structure analyses were intentionally focused on protein sequences encoded by *HLA-DQA1*, whereas sequences encoded by *HLA-DQB1* were not included in further structural comparisons, in accordance with previous structural studies. The analyzed alleles and corresponding protein sequences are summarized in Table 1.

### 2.8. Statistical Analysis

Allelic and genotypic frequencies were calculated using GenePop v4.7 (https://genepop.curtin.edu.au/) [48]. Differences between CD patients and controls were assessed using two-tailed chi-square (χ^2^) tests with Yates’ correction, with statistical significance set at *p* < 0.05. Odds ratios (ORs) and 95% confidence intervals (95% CI) were estimated using OpenEpi v3.01 (https://www.openepi.com/) [49].

## 3. Results

### 3.1. HLA-DRB1 Allele Distribution in CD Patients and Controls

The distribution of HLA-DRB1 alleles was analyzed in 100 patients with CD and 100 healthy controls from the Sardinian population. Allelic frequencies, calculated on the total number of alleles (2n), are summarized in Table 2.

A significantly higher frequency of the predisposing alleles HLA-DRB1*03:01 and HLA-DRB1*04:01 was observed in CD patients compared with controls. Specifically, HLA-DRB1*03:01 accounted for 29.0% of alleles in CD patients versus 9.0% in controls (*p* < 0.001), while HLA-DRB1*04:01 represented 20.5% of alleles in patients compared with 7.5% in controls (*p* < 0.01).

No statistically significant differences were detected for non-predisposing alleles, including HLA-DRB1*01:01, HLA-DRB1*10:01, HLA-DRB1*11:01, and HLA-DRB1*15:01, whose frequencies were comparable between CD patients and controls (Table 2).

### 3.2. HLA-DR/DQ Haplotype Distribution and Genetic Background

To define the genetic framework for subsequent structural analyses, the distribution of HLA-DR/DQ haplotypes was evaluated in CD patients and controls. In the CD cohort, the majority of individuals carried haplotypes including at least one predisposing HLA-DR allele, predominantly DR3–DQ2.5 and, to a lesser extent, DR4–DQ8.

These haplotypes were markedly less frequent in the control group, which showed a higher representation of non-predisposing DR/DQ combinations. In the Sardinian population, the predominance of the DR3–DQ2.5 haplotype reflects the known genetic structure of this island population, characterized by enrichment of specific autoimmune-associated HLA haplotypes and reduced overall haplotypic diversity.

Conversely, the lower frequency of DR4–DQ8 compared with other European populations is consistent with previously reported population-specific differences in HLA class II distribution.

### 3.3. Population-Specific Distribution of HLA-DR/DQ Haplotypes

To contextualize the haplotypic distribution observed in the present cohort, Table 3 provides a comparative overview of major HLA-DR/DQ predisposing haplotypes reported in Caucasian/European and Italian populations, alongside the Sardinian data generated in this study.

Across populations, susceptibility to celiac disease is primarily associated with the HLA-DRB1*03:01–HLA-DQA1*05:01–HLA-DQB1*02:01 haplotype (DQ2.5), which represents the predominant genetic risk configuration in European and Italian cohorts. In the Sardinian cohort, DQ2.5 also emerged as the most prevalent haplotype among CD patients, with frequencies comparable to those reported in other Caucasian populations.

By contrast, the DR4–DQ8 haplotype, which constitutes the second major susceptibility configuration in mainland European populations, was detected at a lower frequency in the Sardinian cohort. The minor-risk DQ2.2 haplotype was identified in a limited number of subjects, whereas non-predisposing HLA-DRB1 alleles were predominantly observed in the control group.

### 3.4. Structural Analysis of HLA Class II Molecules

#### 3.4.1. PSIPRED Prediction of the HLA-DRB1 β1 Domain

**Figure 2:**
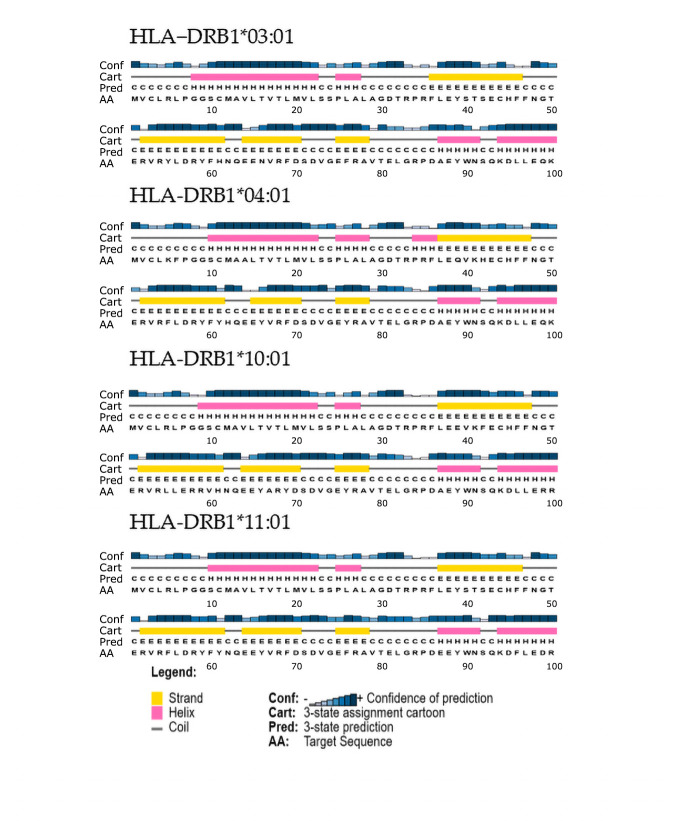
PSIPRED-based secondary structure prediction of the HLA-DRB1 β1 domain. Secondary structure prediction of the β1 domain (approximately residues 1–100) was performed using PSIPRED for predisposing HLA-DRB1 alleles (HLA-DRB1*03:01 and HLA-DRB1*04:01) and non-predisposing reference alleles (HLA-DRB1*10:01 and HLA-DRB1*11:01). Predicted α-helices, β-strands, and coil regions are displayed according to the PSIPRED confidence scale. Predictions are shown as qualitative profiles generated using identical parameters for all analyzed alleles, enabling comparative assessment of overall secondary structure organization and recurrent features, rather than quantitative, residue-level differences. Full-length amino acid sequences of the HLA class II alleles used for the in silico analyses are provided in the Supplementary Materials (Supplementary Figure S1).

PSIPRED-based secondary structure prediction of the HLA-DRB1 β1 domain revealed a highly conserved secondary structure organization across both predisposing alleles (HLA-DRB1*03:01 and HLA-DRB1*04:01) and non-predisposing reference alleles (HLA-DRB1*10:01 and HLA-DRB1*11:01) (Figure 2). The analysis focused on the β1 domain (approximately residues 1–100), which encompasses the structural elements forming the peptide-binding groove of HLA class II molecules.

Across all alleles analyzed, the predicted secondary structure profiles were consistent with the canonical HLA class II fold, characterized by a β-sheet platform forming the floor of the peptide-binding groove and α-helical segments shaping its lateral walls. Comparative inspection identified only subtle, allele-dependent micro-variations in helix continuity and coil distribution, without evidence of alterations in the overall fold or domain organization.

#### 3.4.2. PSIPRED Prediction of the HLA-DQA1 α1 Domain: Structural Organization in DQ2.5 and DQ2.2

Based on the haplotypic framework defined by HLA-DR and HLA-DQ associations, subsequent structural analyses were focused on HLA-DQA1, which encodes the α chain of HLA-DQ heterodimers and contributes to the structural architecture of the peptide-binding groove. PSIPRED-based secondary structure predictions were generated for the full-length HLA-DQA1*05:01 (DQ2.5) and HLA-DQA1*02:01 (DQ2.2) protein sequences.

**Figure 3:**
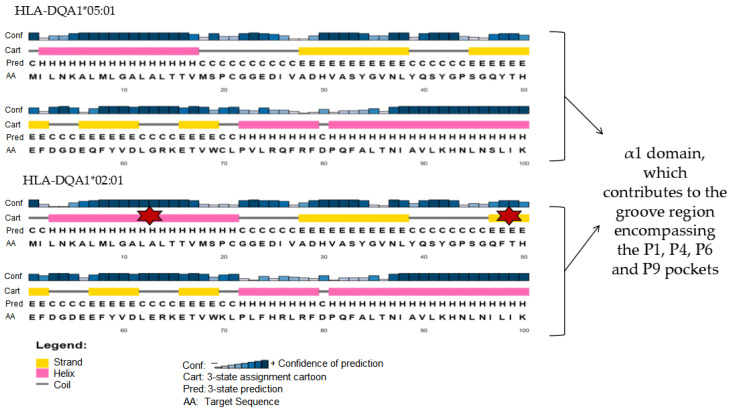
PSIPRED-based secondary structure prediction of the N-terminal α1 domain of HLA-DQA1 contributing to the peptide-binding groove. Secondary structure predictions were generated using PSIPRED for the N-terminal region (approximately residues 1–100) of the HLA-DQA1*05:01 (DQ2.5) and HLA-DQA1*02:01 (DQ2.2) protein sequences. Red asterisks indicate regions displaying the most evident differences in predicted secondary structure organization and are intended solely as visual guides. Predictions are presented as qualitative profiles generated under identical parameters for both alleles, allowing comparative assessment of recurrent structural features rather than quantitative or functional differences. Full-length PSIPRED predictions are provided in the Supplementary Materials (Supplementary Figure S2).

Both alleles displayed the characteristic secondary structure features of HLA class II molecules, consistent with the conserved MHC class II fold. However, comparative analysis revealed allele-dependent differences primarily localized to the N-terminal region of the α1 domain (Figure 3). In particular, the DQ2.5 sequence showed a more coherent and continuous organization of predicted secondary structure elements in this region, whereas the DQ2.2 variant exhibited increased structural discontinuity, reflected by interruptions in helical segments and a higher proportion of coil regions.

Importantly, these differences were confined to localized regions of the α1 domain and did not affect the global secondary structure architecture of the molecule. The observed variations therefore reflect subtle, allele-specific features of secondary structure organization rather than large-scale structural rearrangements.

## 4. Discussion

The present study integrates population-based HLA genotyping with in silico secondary structure analysis to refine the interpretation of HLA class II–associated susceptibility to CD. The genetic analyses delineated a Sardinian haplotypic framework characterized by enrichment of DR3–DQ2.5 and a reduced contribution of DR4–DQ8, consistent with the known population structure of this island cohort. As summarized in Table 3, the overall pattern observed in Sardinia aligns with that reported in European and Italian populations, in which DR3–DQ2.5 represents the predominant susceptibility configuration, whereas DR4–DQ8 and DR7–DQ2.2 account for a smaller proportion of cases [5,9,50,51].

Secondary structure predictions indicated that all analyzed HLA class II molecules display secondary structure profiles consistent with the canonical MHC class II fold that supports antigen presentation. At the level of HLA-DRB1, variations were diffuse and modest (Figure 2), supporting the interpretation that HLA-DR alleles are primarily informative as markers of disease-associated haplotypes rather than as direct mediators of gluten peptide presentation [52]. In this context, the HLA-DR background remains relevant because it defines the linkage framework in which disease-relevant HLA-DQ heterodimers are embedded.

In contrast, analysis of HLA-DQA1 revealed more localized differences within the α1 domain when comparing DQ2.5 and DQ2.2 variants (Figure 3). The more coherent secondary structure organization observed in DQ2.5 provides a structural framework compatible with its stronger genetic association with CD, without implying direct quantitative differences in peptide-binding affinity or functional binding outcomes. These observations are consistent with the concept that allele-dependent structural organization may complement genetic association data by adding an additional interpretative layer at the level of groove-forming regions.

It should be emphasized that PSIPRED-based predictions provide qualitative information on secondary structure propensity rather than quantitative measurements or three-dimensional models [53]. Accordingly, the observed differences reflect reproducible comparative patterns rather than statistically quantified residue-, pocket-, or interaction-level effects. All predictions were generated using identical parameters, ensuring methodological consistency and minimizing subjective interpretation.

Although secondary structure analysis cannot directly model peptide–HLA interactions at atomic resolution, the integration of population-level genetic data with in silico structural features supports the view that CD susceptibility reflects not only the presence of specific HLA alleles and haplotypes but also subtle, allele-specific differences in the organization of groove-forming regions. Homology modeling, molecular docking, and molecular dynamics simulations represent logical next steps to further explore these features in a three-dimensional context, but they were beyond the scope of the present study.

### Clinical Applicability

Genetic testing for HLA class II alleles represents a key component in the diagnostic workup of celiac disease, particularly due to its high negative predictive value. The absence of HLA-DQ2 and HLA-DQ8 strongly argues against the diagnosis, thereby supporting clinical decision-making in patients with equivocal serological, histological, or clinical findings.

Beyond diagnostic exclusion, the present study contributes to a more nuanced interpretation of HLA-associated genetic predisposition. By integrating molecular typing with in silico secondary structure analysis, our results support the concept that HLA-DQ–positive configurations do not necessarily convey equivalent susceptibility. In particular, the comparative differences observed between DQ2.5 and DQ2.2 provide a biologically informed interpretative framework that is consistent with their different strength of association with CD.

For clinicians, this integrated perspective may help contextualize HLA test results, especially in individuals carrying DQ2-related haplotypes who do not develop overt disease. For patients and families, it may facilitate clearer communication that HLA positivity indicates predisposition rather than certainty, helping to reduce misunderstanding and unnecessary anxiety.

In the longer term, structurally informed immunogenetic approaches may contribute to refined interpretative models of genetic susceptibility and support future research efforts aimed at improving risk communication and tailoring follow-up strategies. However, clinical translation will require validation in larger cohorts and functional/structural studies extending beyond secondary structure prediction.

## 5. Conclusions

This study provides an integrated molecular and in silico structural analysis of HLA class II–associated susceptibility to celiac disease in a Sardinian population. By combining population-based HLA genotyping with secondary structure prediction, we define a coherent haplotypic context in which allele-specific structural features can be comparatively interpreted.

While all analyzed HLA class II molecules displayed secondary structure profiles consistent with the canonical architecture supporting antigen presentation, localized differences in secondary structure organization were observed, particularly at the level of HLA-DQ molecules. In this context, the distinct organization detected in DQ2.5 compared with DQ2.2 offers a structurally informed interpretative framework consistent with their different genetic association with celiac disease. Overall, these findings support the value of integrating genetic and structural information to refine the interpretation of HLA-associated disease susceptibility

## Figures and Tables

**Table 1:** HLA class II alleles analyzed in this study. Protein FASTA sequences were retrieved from the IPD–IMGT/HLA database.

Gene	Analyzed Allele	Chain	Associated Haplotype	Biological Role
HLA-DRB1	*03:01	β	DR3–DQ2.5	Predisposing allele within CD-associated haplotypes; β chain of HLA-DR defining the haplotypic background linked to disease-associated HLA-DQ molecules
HLA-DRB1	*04:01	β	DR4–DQ8	Predisposing allele within CD-associated haplotypes; β chain of HLA-DR acting as a marker of the haplotypic background linked to disease-relevant HLA-DQ molecules
HLA-DRB1	*10:01	β	Not associated	Non-predisposing allele; used as structural reference
HLA-DRB1	*11:01	β	Not associated	Non-predisposing allele; used as structural reference
HLA-DQA1	*05:01	α	DQ2.5	α chain of HLA-DQ2.5, major predisposing molecule selected for in silico structural analysis
HLA-DQA1	*02:01	α	DQ2.2	α chain of HLA-DQ2.2, weakly predisposing molecule selected for comparative structural analysis

**Table 2:** Distribution of HLA-DRB1 alleles in CD patients and controls from the Sardinian population. Allelic frequencies are expressed as number and percentage of total alleles.

HLA-DRB1 Allele	CD Patients (2n = 200)	Controls (2n = 200)	*p*-Value
HLA-DRB1*03:01	58 (29.0%)	18 (9.0%)	<0.001
HLA-DRB1*04:01	41 (20.5%)	15 (7.5%)	<0.01
HLA-DRB1*01:01	15 (7.5%)	14 (7.0%)	>0.05
HLA-DRB1*10:01	9 (4.5%)	8 (4.0%)	>0.05
HLA-DRB1*11:01	18 (9.0%)	16 (8.0%)	>0.05
HLA-DRB1*15:01	22 (11.0%)	25 (12.5%)	>0.05

**Table 3:** Comparison of major HLA-DR/DQ predisposing haplotypes across different populations.

Predisposing Allele/Haplotype	European Populations	Italian Population	Sardinian Population (This Study)
HLA-DRB1*03:01/HLA-DQA1*05:01–HLA-DQB1*02:01 (DQ2.5)	~90–95% of CD patients	~85–90% of CD patients	Predominant haplotype (~90% of CD patients)
HLA-DRB1*04:01/HLA-DQA1*03:01–HLA-DQB1*03:02 (DQ8)	~5–10% of CD patients	~6–8% of CD patients	Lower frequency compared with Italian and European cohorts (~5% of CD patients)
HLA-DRB1*07:01/HLA-DQA1*02:01–HLA-DQB1*02:02 (DQ2.2)	2–5% of CD patients; minor risk haplotype	~3–4% of CD patients	Detected in a minority of subjects
Non-predisposing HLA-DRB1 alleles *	~10–15% in healthy controls; rare in CD patients	Similar frequency	Detected mainly in controls; absent or rare in CD patients

* Includes HLA-DRB1 alleles not associated with CD susceptibility, such as HLA-DRB1*10:01 and HLA-DRB1*11:01. Percentages are approximate due to differences in study design and sample size among published cohorts.

## Data Availability

The data presented in this study are available on reasonable request from the corresponding author. The data are not publicly available due to ethical and privacy restrictions.

## Supplementary Materials

The following supporting information can be downloaded at: https://www.mdpi.com/article/10.3390/genes17020145/s1, Figure S1: Full-length amino acid sequences of HLA class II alleles used for in silico analyses. Full-length amino acid sequences of the HLA class II alleles included in this study are shown. The sequences correspond to HLA-DRB1*03:01, HLA-DRB1*04:01, HLA-DRB1*10:01, and HLA-DRB1*11:01 (β chain; 266 amino acids), as well as HLA-DQA1*05:01 and HLA-DQA1*02:01 (α chain; 254 amino acids). All sequences were retrieved from the IPD–IMGT/HLA database, and the corresponding IMGT/HLA accession numbers are reported for each allele. These full-length protein sequences were used as input for secondary structure prediction and subsequent in silico analyses. Figure S2: PSIPRED-based secondary structure prediction of full-length HLA class II molecules analyzed in this study. PSIPRED-based secondary structure predictions of the fulllength HLA class II protein sequences used for in silico analyses are shown. Panels A–D report predictions for the HLA-DRB1 molecules corresponding to the *03:01, *04:01, *10:01, and *11:01 alleles, respectively. Panels E–F show predictions for the HLA-DQA1 molecules corresponding to the *05:01 (DQ2.5) and *02:01 (DQ2.2) alleles. Predicted α-helices, β-strands, and coil regions are displayed according to the PSIPRED confidence scale. These full-length predictions complement the domain-focused analyses presented in Figure 2 and Figure 3.

## Abbreviations

The following abbreviations are used in this manuscript:

CD	Celiac disease
CI	Confidence interval
CLIP	Class II–associated invariant chain peptide
DQ2.2	HLA-DQA1*02:01/HLA-DQB1*02:02 haplotype
DQ2.5	HLA-DQA1*05:01/HLA-DQB1*02:01 haplotype
DQ8	HLA-DQA1*03:01/HLA-DQB1*03:02 haplotype
DR	Human leukocyte antigen DR
HLA	Human leukocyte antigen
IPD–IMGT/HLA	Immuno Polymorphism Database–International ImMunoGeneTics/Human Leukocyte Antigen)
MHC	Major histocompatibility complex
NGS	Next-generation sequencing
OR	Odds ratio
PCR	Polymerase chain reaction
PSIPRED	Protein Structure Prediction Server
SSP	Sequence-specific primers
