# Supplementary material for: Structural Insights into HLA-DQ–Associated Susceptibility to Celiac Disease Through an Integrated Genetic and In Silico Approach in a Sardinian Population
Source: Genes (Basel). 2026 Jan 28;17(2):145. doi: 10.3390/genes17020145 (PMC12940421; doi:10.3390/genes17020145)
Supplement: Supplementary file 1 [file genes-17-00145-s001.zip › genes-4084394-supplementary.pdf]

Figure S1

HLA-DRB1*03:01
IMGT/HLA Acc No: HLA00671

Protein sequence (266 aa)
MVCLRLPGGSCMAVLTVTLMVLSSPLALAGDTRPRFLEYSTSECHFFNGTERVRYLDRYFH
NQEENVRFDSDVGEFRAVTELGRPDAEYWNSQKDLLEQKRGRVDNYCRHNYGVVESFTV
QRRVHPKVTVYPSKTQPLQHHNLLVCSVSGFYPGSIEVRWFRNGQEEKTGVVSTGLIHNG
DWTFQTLVMLETVPRSGEVYTCQVEHPSVTSPLTVEWRARSESAQSKMLSGVGGFVLGLLF
LGAGLFIYFRNQKGHSGLQPRGFLS

HLA-DRB1*04:01
IMGT/HLA Acc No: HLA00685

Protein sequence (266 aa)
MVCLKFPGGSCMAALTVTLMVLSSPLALAGDTRPRFLEQVKHECHFFNGTERVRFLDRYF
YHQEEYVRFDSDVGEYRAVTELGRPDAEYWNSQKDLLEQKRAAVDTYCRHNYGVGESFT
VQRRVYPEVTVYPAKTQPLQHHNLLVCSVNGFYPGSIEVRWFRNGQEEKTGVVSTGLIQN
GDWTFQTLVMLETVPRSGEVYTCQVEHPSLTSPLTVEWRARSESAQSKMLSGVGGFVLGLL
FLGAGLFIYFRNQKGHSGLQPTGFLS

HLA-DRB1*10:01
IMGT/HLA Acc No: HLA00750

Protein sequence (266 aa)
MVCLRLPGGSCMAVLTVTLMVLSSPLALAGDTRPRFLEEVKFECHFFNGTERVRLLERRVH
NQEEYARYDSDVGEYRAVTELGRPDAEYWNSQKDLLERRRAAVDTYCRHNYGVGESFTV
QRRVQPKVTVYPSKTQPLQHHNLLVCSVNGFYPGSIEVRWFRNGQEEKTGVVSTGLIQNG
DWTFQTLVMLETVPQSGEVYTCQVEHPSVMSPLTVEWRARSESAQSKMLSGVGGFVLGLL
FLGAGLFIYFRNQKGHSGLPPTGFLS

HLA-DRB1*11:01
IMGT/HLA Acc No: HLA00751

Protein sequence (266 aa)
MVCLRLPGGSCMAVLTVTLMVLSSPLALAGDTRPRFLEYSTSECHFFNGTERVRFLDRYFY
NQEEYVRFDSDVGEFRAVTELGRPDEEYWNSQKDFLEDRRAAVDTYCRHNYGVGESFTVQ
RRVHPKVTVYPSKTQPLQHHNLLVCSVSGFYPGSIEVRWFRNGQEEKTGVVSTGLIHNGD
WTFQTLVMLETVPRSGEVYTCQVEHPSVTSPLTVEWRARSESAQSKMLSGVGGFVLGLLFL
GAGLFIYFRNQKGHSGLQPRGFLS

HLA-DQA1*05:01
IMGT/HLA Acc No: HLA00613

Protein sequence (254 aa)
MILNKALMLGALALTTVMSPCGGEDIVADHVASYGVNLYQSYGPSGQYTHEFDGDEQFY
VDLGRKETVWCLPVLRQFRFDPQFALTNIAVLKHNLNSLIKRSNSTAATNEVPEVTVFSKSP
VTLGQPNILICLVDNIFPPVVNITWLSNGHSVTEGVSETSFLSKSDHSFFKISYLTLLPSAEESY
DCKVEHWGLDKPLLKHWEPEIPAPMSELTETVVCALGLSVGLVGIVVGTVFIIRGLRSVGAS
RHQGPL

HLA-DQA1*02:01
IMGT/HLA Acc No: HLA00607

Protein sequence (254 aa)
MILNKALMLGALALTTVMSPCGGEDIVADHVASYGVNLYQSYGPSGQFTHEFDGDEEFYV
DLERKETVWKLPLFHRLRFDPQFALTNIAVLKHNLNILIKRSNSTAATNEVPEVTVFSKSPV
TLGQPNTLICLVDNIFPPVVNITWLSNGHSVTEGVSETSFLSKSDHSFFKISYLTFLPSADEIYD
CKVEHWGLDEPLLKHWEPEIPAPMSELTETVVCALGLSVGLVGIVVGTVLIIRGLRSVGASR
HQGPL

Figure S1. Full-length amino acid sequences of HLA class II alleles used for in silico analyses. Full-length amino acid sequences of the HLA class II alleles included in this study are shown. The sequences correspond to HLA-DRB1*03:01, HLA-DRB1*04:01, HLA-DRB1*10:01, and HLA-DRB1*11:01 (β chain; 266 amino acids), as well as HLA-DQA1*05:01 and HLA-DQA1*02:01 (α chain; 254 amino acids). All sequences were retrieved from the IPD–IMGT/HLA database, and the corresponding IMGT/HLA accession numbers are reported for each allele. These full-length protein sequences were used as input for secondary structure prediction and subsequent *in silico* analyses.

Figure S2

A) PSIPRED-based secondary structure prediction of the HLA-DRB1 molecule corresponding to the *03:01 allele

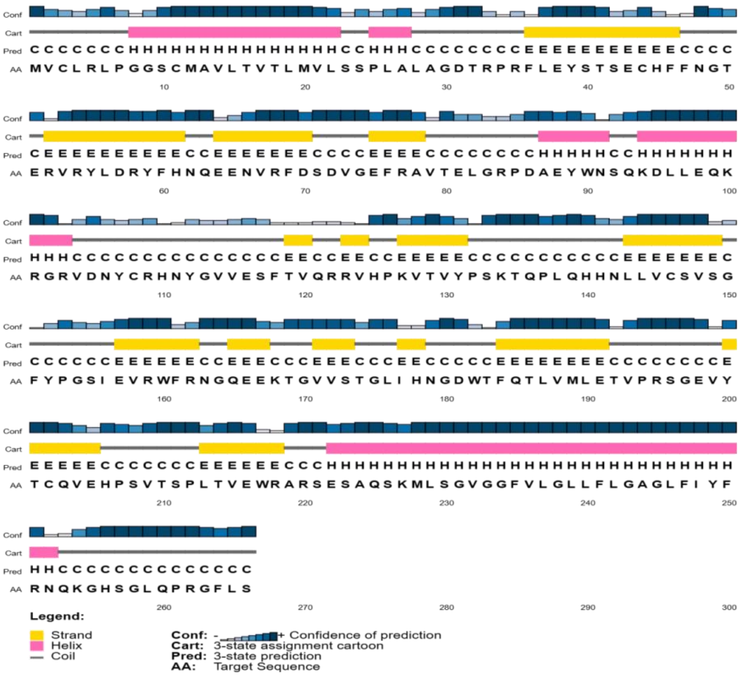

B) PSIPRED-based secondary structure prediction of the HLA-DRB1 molecule corresponding to the *04:01 allele

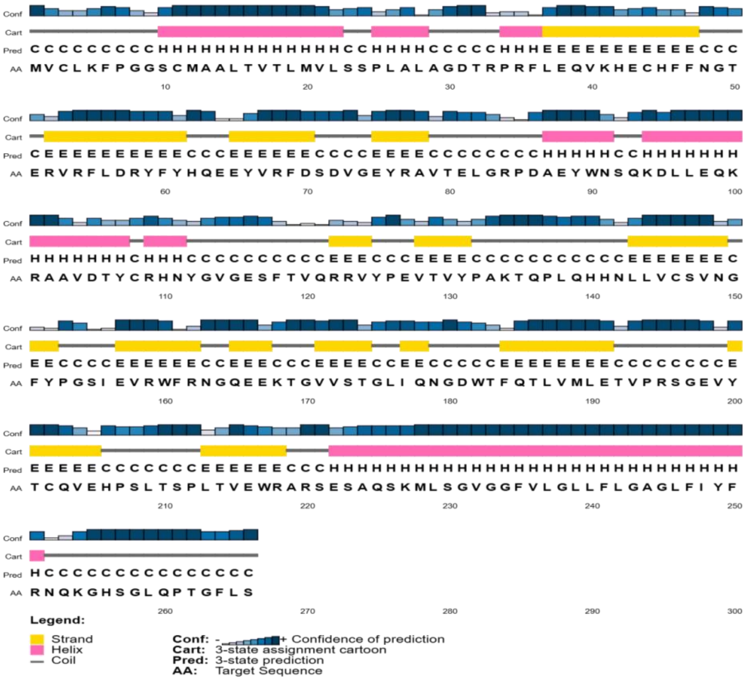

C) PSIPRED-based secondary structure prediction of the HLA-DRB1 molecule corresponding to the *10:01 allele

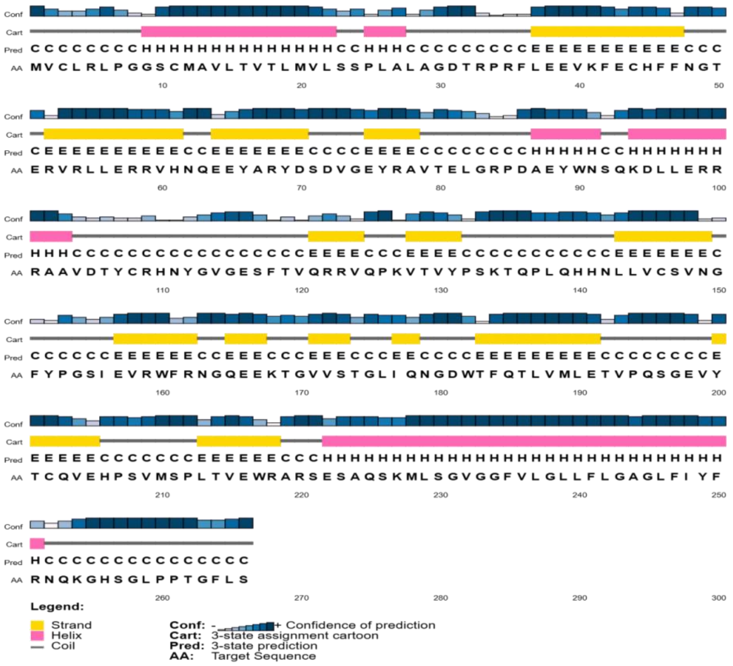

D) PSIPRED-based secondary structure prediction of the HLA-DRB1 molecule corresponding to the *11:01 allele

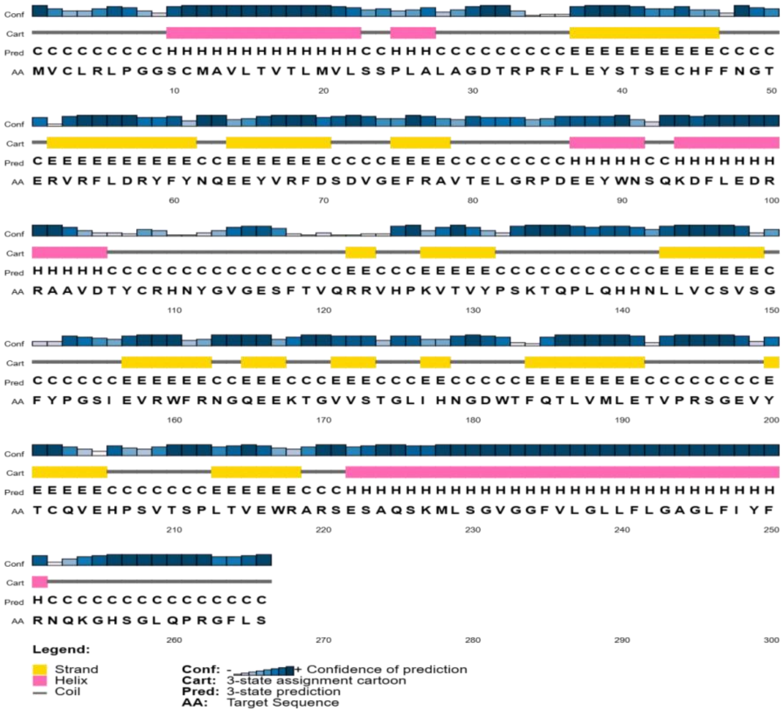

E) PSIPRED-based secondary structure prediction of the HLA-DQA1 molecule corresponding to the *05:01 allele

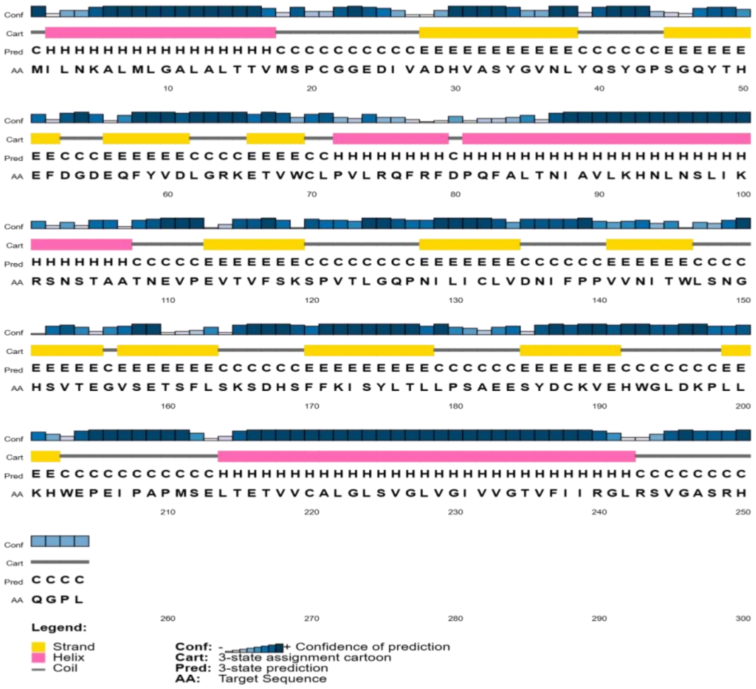

F) PSIPRED-based secondary structure prediction of the HLA-DQA1 molecule corresponding to the *02:01 allele

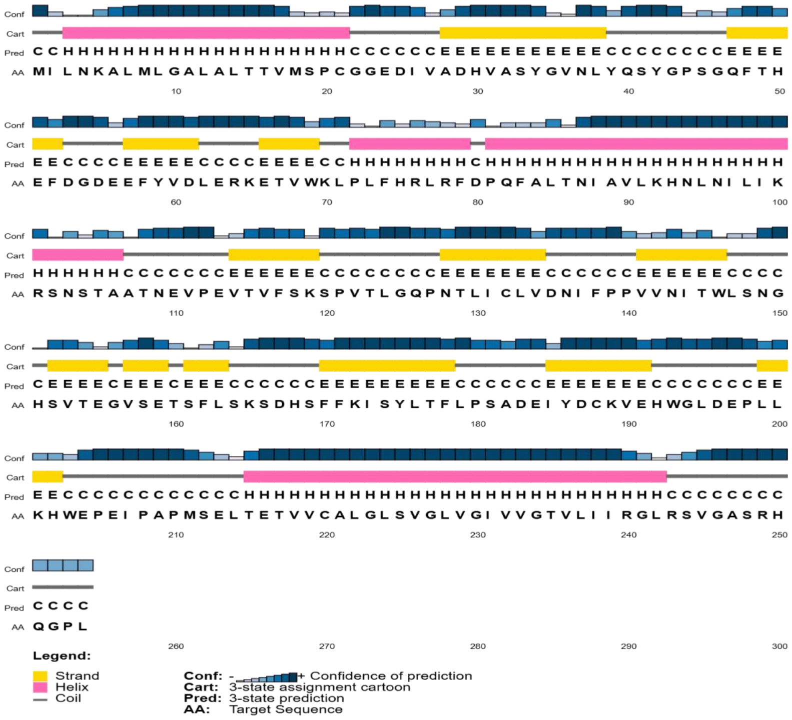

Figure S2. PSIPRED-based secondary structure prediction of full-length HLA class II molecules analyzed in this study. PSIPRED-based secondary structure predictions of the full-length HLA class II protein sequences used for in silico analyses are shown. Panels A–D report predictions for the HLA-DRB1 molecules corresponding to the *03:01, *04:01, *10:01, and *11:01 alleles, respectively. Panels E–F show predictions for the HLA-DQA1 molecules corresponding to the *05:01 (DQ2.5) and *02:01 (DQ2.2) alleles. Predicted α-helices, β-strands, and coil regions are displayed according to the PSIPRED confidence scale. These full-length predictions complement the domain-focused analyses presented in Figures 2 and 3.
